# Enhanced visible-light photocatalytic activity and photostability of Ag_3_PO_4_/Bi_2_WO_6_ heterostructures toward organic pollutant degradation and plasmonic Z-scheme mechanism†

**DOI:** 10.1039/c8ra01477a

**Published:** 2018-04-27

**Authors:** Fengyan Ma, Qilin Yang, Zhengjun Wang, Yahong Liu, Jianjiao Xin, Jingjing Zhang, Yuting Hao, Li Li

**Affiliations:** College of Chemistry and Chemical Engineering, Qiqihar University Qiqihar 161006 Heilongjiang P. R. China; College of Materials Science and Engineering, Qiqihar University Qiqihar 161006 Heilongjiang P. R. China qqhrlili@126.com

## Abstract

Novel Ag_3_PO_4_/Bi_2_WO_6_ heterostructured materials with enhanced visible-light catalytic performance were successfully synthesized by assembly combined with a hydrothermal treatment. The microstructures, morphologies, and optical properties of the prepared samples were characterized by multiple techniques. The irregular Ag_3_PO_4_ nanospheres dispersed on the surface of Bi_2_WO_6_ nanoflakes, and their catalytic performances were evaluated *via* the degradation of organic pollutants including rhodamine B (RB), methylene blue (MB), crystal violet (CV), methyl orange (MO), and phenol (Phen) under visible-light irradiation. The resulting Ag_3_PO_4_/Bi_2_WO_6_ heterostructured materials displayed higher photocatalytic activity than that of either pure Bi_2_WO_6_ or Ag_3_PO_4_. The enhanced photocatalytic activity was due to the good formation of heterostructures, which could not only broaden the spectral response range to visible light but also effectively promoted the charge separation. Meanwhile, the reasonable photoreactive plasmonic Z-scheme mechanism was carefully investigated on the basic of the reactive species scavenging tests, photoelectrochemical experiments, and photoluminescence (PL) spectrum. In addition, the excellent photostability of Ag_3_PO_4_/Bi_2_WO_6_ was obtained, which Ag formed at the early photocatalytic reaction acted as the charge transmission-bridge to restrain the further photoreduction of Ag_3_PO_4_.

## Abstract 2

Novel Ag_3_PO_4_/Bi_2_WO_6_ heterostructured materials were synthesized by assembling Ag_3_PO_4_ irregular nanospheres on the surface of Bi_2_WO_6_ nanoflakes and showed superior visible-light photocatalytic activity owing to the good formation of heterostructures.

## Graphical abstract

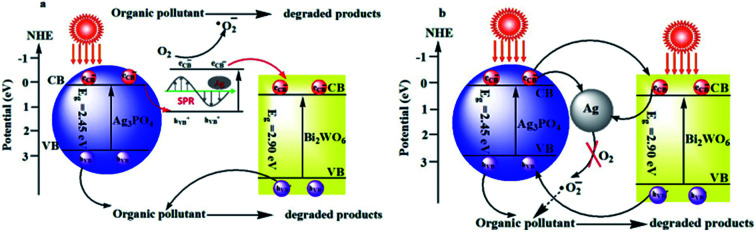

## 1. Instruction

Recently, semiconductor photocatalysis has attracted more and more attention as a green, highly efficient technology for resolving the current energy and environmental problems.^1–3^ Photocatalysts based on TiO_2_, ZnO are inactive under visible light irradiation due to their wide bandgap energy and improper band position, which requires ultraviolet irradiation that only accounts for less than 5% of the solar energy.^4–6^ And the main reason for their poor photocatalytic activity might be due to the strong recombination of photogenerated electron–hole (e^−^–h^+^) pairs, which is a widespread phenomenon among semiconductor photocatalysts. Therefore, it is still challenging to extend the photo-response range and improve the charge separation efficiency of the semiconductor photocatalysts for satisfying the requirements of applications.

Recently, Bi-based semiconductor materials, such as Bi_2_WO_6_, BiVO_4_, Bi_2_MO_6_, Bi_2_Mo_2_O_9_, Bi_24_O_31_Br_10_, and BiOBr, have attracted widespread attention in photocatalytic application owing to the relatively narrow gap, nontoxicity, chemical inertness, stability, and sunlight utilization for wastewater treatment.^7–14^ Among the Bi-based semiconductor materials, Bi_2_WO_6_, as a typical aurivillius oxide, is regarded one of the ideal photocatalyst materials because of its unique layered structure feature and relatively high visible photocatalytic activity. However, the photocatalytic activity of bare Bi_2_WO_6_ is limited by the high recombination rate of photogenerated e^−^–h^+^ pairs and the low absorption capacity of visible-light (less than 450 nm).^15,16^ Therefore, how to improve separation efficiency of photoinduced e^−^–h^+^ pairs during the photocatalytic reactions becomes a key issue for developing the Bi_2_WO_6_-based photocatalysts.

More recently, among the reported Ag-based semiconductors, especially silver orthophosphate (Ag_3_PO_4_), which has a strong absorption (*λ* < 530 nm), has been considered as a promising photocatalyst with outstanding visible-light catalytic activity.^17,18^ Meanwhile, Ag_3_PO_4_ possesses extremely oxidation power, which not only oxidizes H_2_O to produce O_2_ but also degrades organic pollutants under visible-light irradiation.^19–22^ However, there are some flaws limit its extensive use as following: (1) Ag_3_PO_4_ is slightly soluble in water due to its larger solubility product constant (*K*_sp_ = 1.6 × 10^−16^)^23^; (2) as the photosensitive material, it can be easily corroded by light and reduced to Ag^0^ in the photocatalytic process without electron acceptors;^24^ (3) it suffers from the fast recombination of photogenerated charge-carries.^25^

In the present study, Ag_3_PO_4_ is introduced on the basis of the following points: (1) Ag_3_PO_4_ is a narrow-semiconductor (*E*_g_ = 2.4 eV), and its introduction is beneficial to extend the light response of Bi_2_WO_6_ to a higher wavelength region and thereby decreasing its band gap; (2) two semiconductors possess the matching band structure, similar band gap, and the proper molar ratio, and build effective heterostructures, which promote the efficient separation of the e^−^–h^+^ pairs and therefore increasing the quantum yield; (3) part Ag_3_PO_4_ is reduced to metallic Ag during the initial photocatalytic reaction. It is precisely this part of the elemental silver can be used as a solid-state electron mediator to accelerate charge separation through Z-scheme system, which improve the charge separation, prevent the continuing reductive decomposition of Ag_3_PO_4_, and enhance its stability. Hence, the coupling of Bi_2_WO_6_ with Ag_3_PO_4_ to form heterostructures is considered to be one of the most promising methods to improve the visible-light-driven catalytic performance of photocatalysts (Bi_2_WO_6_ or Ag_3_PO_4_) because the heterostructures not only broaden the spectral response range to visible light but also promote the charge separation. This point is confirmed by the deposition of Ag_3_PO_4_ onto certain semiconductor materials. Please see the Table 1 (ESI†). Table 1 summarizes the detailed information on literature reported Ag_3_PO_4_ photocatalysts. Compared with the semiconductor materials, such as ZnO,^26^ graphene^27^ (G), InVO_4_/BiVO_4_,^28^ and Bi_2_MoO_6_,^29^ Bi_2_WO_6_ as support can well improve the photocatalytic activity of Ag_3_PO_4._

**Table 1:** Textural parameters of various materials

Samples	*S*_BET_ (m^2^ g^−1^)	*V*_p_ (cm^3^ g^−1^)	*D*_p_ (nm)
Ag_3_PO_4_	8.06	0.038	11.47
Ag_3_PO_4_/Bi_2_WO_6_-0.3	14.50	0.049	9.46
Ag_3_PO_4_/Bi_2_WO_6_-0.5	18.21	0.070	10.74
Bi_2_WO_6_	43.55	0.16	10.44

Among a good deal of the heterojunction photocatalysts, the all-solid-state Z-scheme photocatalytic system possesses advantages over improving separation ratio of photo-induced e^−^–h^+^ pairs and enhancing the stability of photocatalyst. In general, the all-solid-state Z-scheme photocatalytic system consists of two different semiconductor materials and an electron mediator. Notably, metallic Ag as a solid-state electron mediator would contribute to the construction of Z-scheme system. Recently, Yuan *et al.* successfully prepared Ag_3_PO_4_/CuBi_2_O_4_ photocatalyst and formed Ag_3_PO_4_/Ag/CuBi_2_O_4_ photocatalyst during the photocatalytic degradation process, which can enhance photocatalytic activity and stability of photocatalyst through the formation of Z-scheme system.^30^ Liu *et al.* also designed a novel visible-light-driven Ag/Ag_3_PO_4_/WO_3_ Z-scheme heterostructures by a facile deposition–precipitation method followed by photoreducing Ag^+^ into metallic Ag. The Ag/Ag_3_PO_4_/WO_3_ showed enhanced photocatalytic RhB efficiency, indicating the formed Z-scheme heterostructures could efficiently promote the separation and transfer of photogenerated e^−^–h^+^ pairs.^31^ So the combination of Bi_2_WO_6_ and Ag_3_PO_4_ to construct Z-scheme would be an efficient strategy.

To the best of our knowledge, there is less report on the achievement of Bi_2_WO_6_-based heterojunction by the introduction of Ag_3_PO_4_. For instance, Somchai Thongtem prepared Ag_3_PO_4_/Bi_2_WO_6_ toward RB (5 ppm) degradation efficiency can reach 100% after 80 min visible-light illumination.^32^ Because it is still not sufficient for practical application. It is necessary to further improve the photocatalytic activity of Ag_3_PO_4_/Bi_2_WO_6_. In addition, there is no corresponding research reports to discuss the Ag_3_PO_4_/Bi_2_WO_6_ for pollutants' removal based on the plasmonic Z-scheme mechanism. Therefore, it is meaningful to apply the SPR effect of Ag nanoparticles into the hybrid composite to design highly efficient visible-light-driven photocatalysts based on Z-scheme charge transfer mechanism.

Herein, we successfully fabricated Ag_3_PO_4_/Bi_2_WO_6_ heterostructures *via* the assembly combined with a hydrothermal technique. The Ag_3_PO_4_/Bi_2_WO_6_ heterostructures displayed enhanced photocatalytic performance toward organic pollutants (including rhodamine B (RB), phenol (Phen), methyl orange (MO), crystal violet (CV), and methylene blue (MB)) degradation under visible-light irradiation. Subsequently, microstructures, surface chemical states, specific surface areas, optical, and photoelectrochemical properties of Ag_3_PO_4_/Bi_2_WO_6_ heterojunction were systematically studied. Meanwhile, a possible visible-light-induced photocatalytic mechanism of Ag_3_PO_4_/Bi_2_WO_6_ heterostructures was proposed.

## 2. Experiment section

### 2.1 Preparation of photocatalysts

#### 2.1.1 Preparation of Bi_2_WO_6_

All the reagents used in the experiment were of analytic grades, commercially purchased and used without further purification. In a typical process, 0.972 g Bi(NO_3_)_3_ and 0.329 g Na_2_WO_4_ were dissolved in glacial acetic acid (10 mL) and H_2_O (10 mL), respectively. Then the Na_2_WO_4_ solution was dropwise added into Bi(NO_3_)_3_ solution. After being continuously stirred for 4 h, the mixture was transferred to Teflon-coated autoclave and held at 150 °C for 20 h followed by cooling at room temperature naturally. To remove any residue of by-products and reactants, the obtained Bi_2_WO_6_ was washed with deionized water several times and dried at 80 °C for 24 h.

#### 2.1.2 Preparation of Ag_3_PO_4_/Bi_2_WO_6_

Ag_3_PO_4_/Bi_2_WO_6_ composites with the different molar ratio were synthesized as follows. 1.092 g AgNO_3_ and 0.762 g Na_3_PO_4_ were dissolved in deionized water (10 mL) and stirred for 30 min, respectively. Then Na_3_PO_4_ solution was dropwise added into the AgNO_3_ solution to form yellow sediments with stirring for 2 h. Subsequently, a certain amount of as-prepared Bi_2_WO_6_ was slowly added to the above mixture and stirred for 4 h followed by drying at 60 °C for 24 h. Ultimately, the obtained Ag_3_PO_4_/Bi_2_WO_6_ samples were collected by washing, filtration, and drying at 60 °C for 48 h. The series of photocatalysts prepared were labeled as Ag_3_PO_4_/Bi_2_WO_6_-*x*, where *x* presents the molar ratio of Bi_2_WO_6_ to Ag_3_PO_4_. Pure Ag_3_PO_4_ was acquired in the absence of Bi_2_WO_6_ through the same process.

### 2.2 Characterization of photocatalysts

The crystal structures were obtained on a Bruker-AXS (D8) X-ray diffractometer with Cu Kα radiation. X-ray photoelectron spectroscopy (XPS) characterization was carried out on an ESCALAB 250Xi spectrometer equipped with Al Kα radiation at 300 W. N_2_ adsorption–desorption isotherm analysis of samples were obtained at 77 K using Micromeritics 3H-2000PS2. The morphologies of synthesized samples were analyzed using a scanning electron microscope (SEM) (Hitachi S-4300) and transmission electron microscope (TEM) and high resolution transmission electron microscope (HRTEM) (JEM-2100F). The UV-visible diffuse reflectance spectra (UV-vis-DRS) were recorded using a UV-vis spectrophotometer (TU-1901) over the wavelength range of 200–800 nm using BaSO_4_ as the reflectance standard material. Fourier transform infrared (FT-IR) spectra were recorded using an FT-IR spectrophotometer (PE Company, America). Photoluminescence spectra (PL) were obtained by a Hitachi F-7000 spectrofluorometer with an excitation wavelength of 360 nm, and all the samples were pressed into pellets in the sample holder.

### 2.3 Photocatalytic tests

Photocatalytic activities of the Ag_3_PO_4_/Bi_2_WO_6_ heterostructures were studied by monitoring the degradation behaviors of organic contaminants, including RB, Phen, MO, CV, and MB. The photocatalytic experiments were carried out in a hollow cylindrical photoreactor equipped with a water jacket. 400 W Xe lamp (*λ* > 410.0 nm; moreover, the inner sleeve was made of No. 11 glasses to filter out ultraviolet from the Xe lamp) was used as the visible-light source. The Xe lamp was positioned within the inner part of the photoreactor and cooling water was circulated through a pyrex jacket surrounding the lamp to keep room temperature. In a typical experimental procedure, 200 mg of catalyst was placed into 220 mL of dye (50 ppm) or Phen (25 ppm) solution *via* ultrasonication for 10 min, and magnetically stirred in the dark for 1 h to establish the adsorption–desorption equilibrium between the catalyst and organic contaminant. Then the suspension was exposed to visible light irradiation under magnetic stirring. 4 mL of the suspension was collected at a regular time interval and analyzed after centrifugation. The dye concentration was analyzed by UV-vis spectrophotometer (TU-1901) at the maximum absorption spectra. Changes of Phen concentrations were monitored by a Yilite P230II HPLC: C_18_ column, UV detector (*λ* = 270 nm), methanol/water (50/50, v/v), and 1 mL min^−1^.

### 2.4 Photoelectrochemical experiments

Electrochemical measurements were carried out in a traditional three-electrode system (CHI660E, China). Indium-tin oxide (ITO) glass electrode (1 cm^2^), saturated calomel electrode (SCE), and Pt sheet were used as the working electrode, reference electrode, and the counter electrode, respectively. The sample mixed with Nafion ionomer was dissolved ethanol aqueous solution to obtain 5 mg L^−1^ suspension. And then the suspension was uniformly drop-coated onto the clean ITO electrode surface and dried in air. A Xe lamp was used as the light source, and aqueous Na_2_SO_4_ solution (0.01 mol L^−1^) served as the electrolyte. All the experiments were performed at room temperature (about 25 °C). The photocurrent was measured under light illumination from a 400 W Xe lamp. The electrochemical impedance study was carried out over a frequency domain of 1 Hz to 100 kHz with a sinusoidal perturbation potential of 5 mV.

## 3. Results and discussion

### 3.1 Characteristics of microstructures of Ag_3_PO_4_/Bi_2_WO_6_

**Fig. 1:**
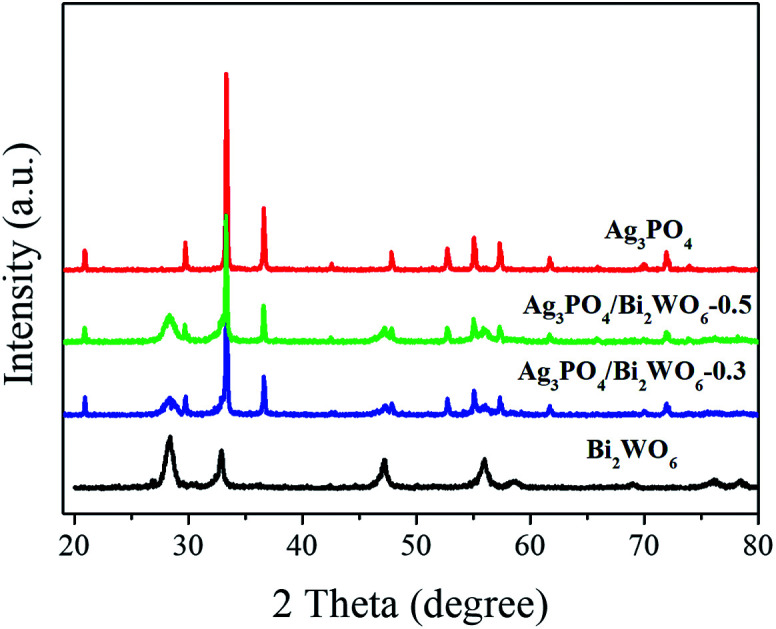
XRD patterns of the pristine Bi_2_WO_6_, Ag_3_PO_4_, and Ag_3_PO_4_/Bi_2_WO_6_ composites.

XRD is used to investigate the crystal structure of the samples. As displayed in Fig. 1, the distinct diffraction peaks of Bi_2_WO_6_ can be found 28.3°, 32.8°/32.9°, 47.1°, 56.0°, 58.5°, 68.8°, 75.9°, and 78.5°, which can be attributed to the (131), (200)/(002), (202), (133), (262), (400), (193), and (204) crystal planes of orthorhombic phase of Bi_2_WO_6_ (JCPDS 39-0256). While for pure Ag_3_PO_4_, the characteristic peaks of 21.7° (110), 29.7° (200), 33.3° (210), 36.5° (211), 47.8° (310), 52.6°(222), 54.9°(320), 57.2° (321), 61.8°(400), and 71.8°(421) are well indexed as the body-centered cubic phase of Ag_3_PO_4_ (JCPDS 06-0505). In case of Ag_3_PO_4_/Bi_2_WO_6_ composites, they show the coexistence of Bi_2_WO_6_ phase and Ag_3_PO_4_ phase. Furthermore, the peak intensity of Bi_2_WO_6_ increases with increasing the Bi_2_WO_6_ loading, while the peak intensity of Ag_3_PO_4_ lowers simultaneously. However, the peak position of Ag_3_PO_4_ does not significantly change, which indicates Bi_2_WO_6_ is not incorporated into Ag_3_PO_4_ lattice. In addition, no diffraction peaks of Ag or other impurities are found.

**Fig. 2:**
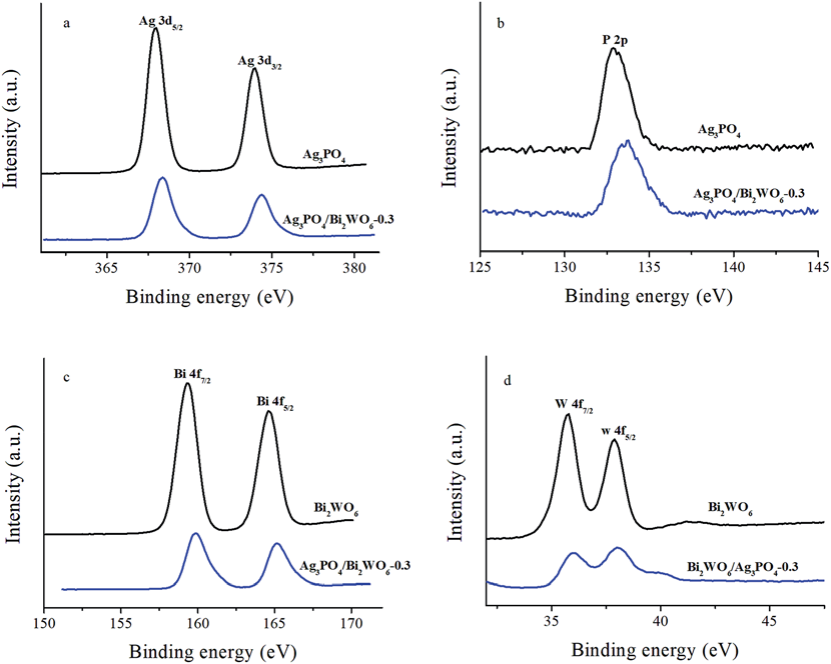
The high-resolution XPS spectra of Ag 3d (a), P 2p (b), Bi 4f (c), and W 4f (d) for the Ag_3_PO_4_, Bi_2_WO_6_, and Ag_3_PO_4_/Bi_2_WO_6_-0.3.

X-ray photoelectron spectroscopy (XPS) as surface analytic technique is employed to detect the electronic structures of Ag_3_PO_4_, Bi_2_WO_6_, and Ag_3_PO_4_/Bi_2_WO_6_ composites. Fig. 2a shows the high-resolution XPS spectra of Ag of the Ag_3_PO_4_ and Ag_3_PO_4_/Bi_2_WO_6_ composite catalysts. The typical peaks of Ag 3d at 368.0 eV (Ag 3d_5/2_) and 374.0 eV (Ag 3d_3/2_) are ascribed to Ag^+^ in Ag_3_PO_4_.^33^ After the Bi_2_WO_6_ introduction, the binding energy of spin-obit Ag 3d is divided into two peaks at 368.3 and 374.3 eV (ref. 24 and 34), which is 0.3 eV higher than those of Ag_3_PO_4_. The characteristic peaks of Ag nanocrystals existing on the surface of Ag_3_PO_4_ and Ag_3_PO_4_/Bi_2_WO_6_ are not found, suggesting no Ag^0^ was formed during the preparation of the catalysts. Compared with pure Ag_3_PO_4_, the binding energy of P 2p of Ag_3_PO_4_/Bi_2_WO_6_ is turned from 132.8 eV to a higher value of 133.8 eV (Fig. 2b). As shown in Fig. 2c, the peaks located at 159.3 eV, 164.6 eV, and 159.9 eV, 165.2 eV correspond to the Bi 4f_7/2_ and Bi 4f_5/2_ of Bi_2_WO_6_ and Ag_3_PO_4_/Bi_2_WO_6_, respectively, implying the bismuth species in the composite is Bi^3+^ cations. The peaks for W 4f_7/2_ (35.98 eV) and W 4f_5/2_ (37.98 eV) can be attributed to a six-valent oxidation state for W^6+^ in Ag_3_PO_4_/Bi_2_WO_6_, meaning 0.2 and 0.1 eV deviation of 4f_7/2_ and 4f_5/2_ relative to the values in pure Bi_2_WO_6_ (Fig. 2d).

The O 1s high-resolution XPS spectra of samples are provided in Fig. S1 (ESI†). The O 1s XPS spectra of Bi_2_WO_6_ (Ag_3_PO_4_) show three individual peaks with the binding energies of 530.2 eV (530.2 eV, denoted as O 1s(1)), 531.6 eV (531.3 eV, denoted as O 1s(2)), and 533.1 eV (533.1 eV, denoted as O 1s(3)), which can be attributed to the lattice oxygen in Bi_2_WO_6_ (Ag_3_PO_4_), the external hydroxyl groups, and adsorbed oxygen species at the surface of the composite.^35–37^ For Ag_3_PO_4_/Bi_2_WO_6_-0.3, the binding energies of O 1s orbits are 529.9 eV, 531.4 eV, and 533.1 eV, respectively. And they also display more or less lower energy shift compared with individual counterparts. The variations of the binding energies of Ag 3d, P 2p, Bi 4f, W 4f, and O 1s are attributed to chemical interactions between Bi_2_WO_6_ and Ag_3_PO_4_, which proves the formation of heterostructures and facilitates interfacial charge transfer, leading to the improved photocatalytic activity of Ag_3_PO_4_/Bi_2_WO_6_ nanocomposites. Beyond that, the tight chemical interactions can improve the structural stability of photocatalyst. The similar results were also reported by another group.^38,39^

**Fig. 3:**
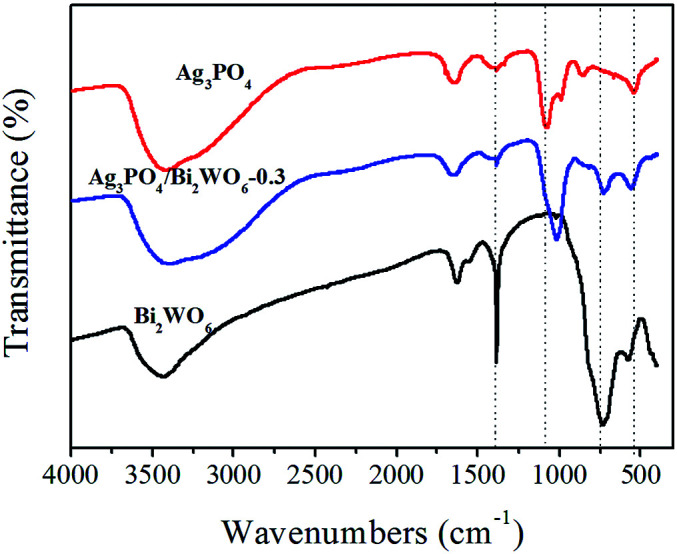
FTIR spectra of Bi_2_WO_6_, Ag_3_PO_4_, and Ag_3_PO_4_/Bi_2_WO_6_.

FT-IR spectra were carried out to investigate the presence of Ag_3_PO_4_ and Bi_2_WO_6_ in the Ag_3_PO_4_/Bi_2_WO_6_ composite. As shown in Fig. 3, the typical peaks located at 577.8 cm^−1^, 731.6 cm^−1^, and 1384 cm^−1^ are attributed to the Bi–O, W–O, and W–O–W bridging stretching modes, respectively, which indicates the existence of Bi_2_WO_6_.^40,41^ For pure Ag_3_PO_4_, the stronger peak at 543.1 cm^−1^ is ascribed to bending vibration of O=P–O. The other absorption bands at 859.9 cm^−1^ and 1076.4 cm^−1^ are assigned to the symmetric and asymmetric stretching vibrations of P–O–P rings.^42^ After the Bi_2_WO_6_ introduction, the typical peaks corresponding Bi–O, W–O, and W–O–W stretching modes in Ag_3_PO_4_/Bi_2_WO_6_ composite disappear, shift to lower value, and become weak, respectively. However, compared with pure Ag_3_PO_4_, the wavenumbers of P–O–P symmetric and asymmetric stretching vibrations shift to lower values of 817.6 cm^−1^ and 1017.5 cm^−1^. Meanwhile, that of O=P–O changes from 543.1 cm^−1^ to 559.3 cm^−1^. In addition, the bands at 3400 cm^−1^ and 1654 cm^−1^ are related to the –OH stretching and vibration of adsorbed H_2_O on the surface of photocatalysts. These results further verify the interaction between Bi_2_WO_6_ and Ag_3_PO_4_, meaning Bi_2_WO_6_ was successfully modified Ag_3_PO_4_.

**Fig. 4:**
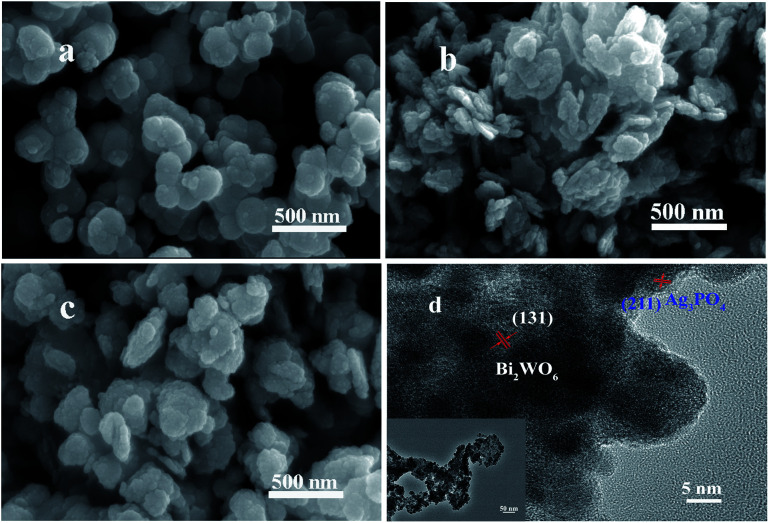
SEM images of Ag_3_PO_4_ (a), Bi_2_WO_6_ (b), Ag_3_PO_4_/Bi_2_WO_6_-0.3 (c), and HRTEM (d) and TEM images (inset d) of Ag_3_PO_4_/Bi_2_WO_6_-0.3.

The typical morphologies of Ag_3_PO_4_, Bi_2_WO_6_, and Ag_3_PO_4_/Bi_2_WO_6_ composites were characterized by SEM images. From Fig. 4a, Ag_3_PO_4_ shows irregular spherical morphology with a diameter of 100–180 nm. While Bi_2_WO_6_ exhibits a typical structure of nanoflakes consist of nanoparticles with the side length of 50–250 nm (Fig. 4b). Fig. 4c illustrates the typical SEM image of Ag_3_PO_4_/Bi_2_WO_6_-0.3, where irregular spherical Ag_3_PO_4_ nanoparticles disperse on the surface of Bi_2_WO_6_ nanoflakes. These tiny particles intertwine with each other, suggesting that Ag_3_PO_4_ nanoparticles can restrain the agglomeration of Bi_2_WO_6_ nanoflakes.

The morphological and microstructural details of Ag_3_PO_4_/Bi_2_WO_6_-0.3 are obtained by TEM and HRTEM technique. As displayed in the inset of Fig. 4d, the Ag_3_PO_4_ irregular spheres disperse over the surface of Bi_2_WO_6_ nanoflakes, which is coincided with the aforementioned SEM observations. From the HRTEM image of Ag_3_PO_4_/Bi_2_WO_6_-0.3 (Fig. 4d), the lattice distances for the Bi_2_WO_6_ (131) and Ag_3_PO_4_ (211) facets are measured to be 0.315 and 0.245 nm, respectively, which is in well accordance with the XRD results. Furthermore, to further prove the formation of heterostructure, the energy dispersive spectroscopy (EDS) elemental mapping of Ag_3_PO_4_/Bi_2_WO_6_-0.3 was performed. As shown in Fig. S2(b–f) (ESI†), Ag_3_PO_4_ and Bi_2_WO_6_ are relatively uniform dispersion and well connected, which is in accordance with that of SEM and TEM images. Therefore, according to the above results, it gives solid evidence for the formation of heterostructure between Bi_2_WO_6_ and Ag_3_PO_4_.

**Fig. 5:**
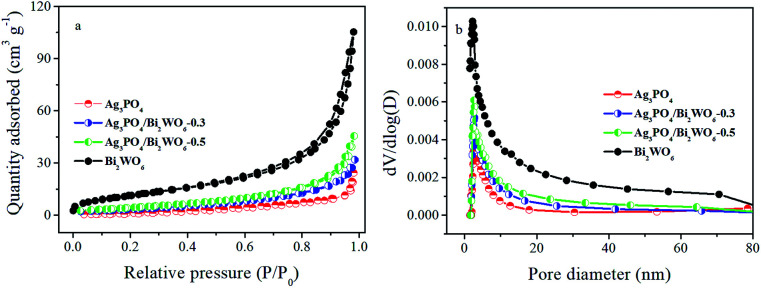
N_2_ adsorption–desorption isotherm curves (a) and pore size distribution (b) of the as-prepared samples.

The BET specific surface area and the porous structure of the samples were analyzed by nitrogen adsorption–desorption isotherms, the results shown in Table 1. As shown in Fig. 5a, the N_2_ isotherms of samples show characteristic type IV isotherms with H3 hysteresis loops, implying the presence of mesopores in the size of 2–50 nm. This result can be further proved by the pore size distribution analysis (Fig. 5b). And the specific surface area of pure Ag_3_PO_4_, Ag_3_PO_4_/Bi_2_WO_6_-0.3, Ag_3_PO_4_/Bi_2_WO_6_-0.5, and Bi_2_WO_6_ is 8.06, 14.50, 18.21, and 43.55 m^2^ g^−1^, respectively. The BJH absorption cumulative pore volume increases from 0.038 to 0.070 cm^3^ g^−1^ after incorporation with Bi_2_WO_6_. The results imply the specific surface area and pore volume of Ag_3_PO_4_/Bi_2_WO_6_ slight enhance with increasing Bi_2_WO_6_ loading contrast with Ag_3_PO_4_. But the specific surface area and pore volume have no obvious change among these samples, which play a minor role in the enhanced photocatalytic activity of Ag_3_PO_4_/Bi_2_WO_6_ composite.

### 3.2 Optical absorption properties

**Fig. 6:**
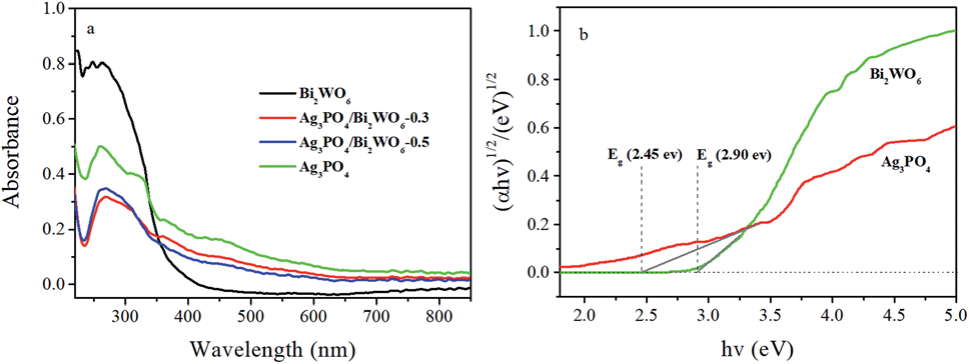
UV-vis DRS (a) and the plots of (*αhν*)^1/2^*versus* photon energy for the band gap energies (b) of Ag_3_PO_4_, Bi_2_WO_6_, and Ag_3_PO_4_/Bi_2_WO_6_ photocatalysts.

The optical absorbance properties and gap energies of the Ag_3_PO_4_, Bi_2_WO_6_, and Ag_3_PO_4_/Bi_2_WO_6_ composites were determined using the UV-vis-DRS, and the results were displayed in Fig. 6. From Fig. 6a, the pure Bi_2_WO_6_ exhibits strong absorbance in the wavelengths shorter than 450 nm owing to the intrinsic band-gap transition,^43^ and Ag_3_PO_4_ has the absorption edge at about 530 nm, which is consistent with the reported result.^44^ Compared with Bi_2_WO_6_, the wavelength regions of the Ag_3_PO_4_/Bi_2_WO_6_ composite are extended towards the visible-light region. Moreover, the more obvious red shift phenomenon is observed with increasing the Ag_3_PO_4_ loading, which implies more visible light is absorbed by the composite, and produce more e^−^–h^+^ pairs, further improve the photocatalytic activity.

The band gap energies of the photocatalysts are calculated according to the following equation:

1 (*αhν*) = *A* (*hν* − *E*_g_)^*n*/2^

In this equation, *A*, *α*, *h*, *hν*, and *E*_g_ are constant, absorption coefficient, Planck constant, the energy of the incident photon, and band gap, respectively. And *n* is 1 and 4 for a direct and indirect band gap semiconductor, respectively. The value of *n* for Bi_2_WO_6_ and Ag_3_PO_4_ is 1 (ref. 20 and 45). By calculating, the band gaps of Bi_2_WO_6_ and Ag_3_PO_4_ are 2.90 and 2.45 eV, respectively. The band edge positions of photocatalysts can be determined by the empirical equations:

2 *E*_VB_ = *X* − *E*^e^ + 0.5 *E*_g_

3 *E*_CB_ = *E*_VB_ − *E*_g,_

where *E*_VB_ is the valence band edge potential, *E*_CB_ is the conduction band edge potential, *X* is the electronegativity of the semiconductor obtained from the geometric mean of the electronegativity for the component atoms (6.21 eV for Bi_2_WO_6_ and 5.965 eV for Ag_3_PO_4_), *E*^e^ is the energy of free electrons on the hydrogen scale (4.5 eV), *E*_g_ is the band gap energy of the semiconductor. Thus, *E*_VB_ and *E*_CB_ are calculated to be 3.34 eV and 0.44 eV for Bi_2_WO_6_ and 2.69 eV and 0.24 eV for Ag_3_PO_4_, respectively.

### 3.3 Photocatalytic tests

**Fig. 7:**
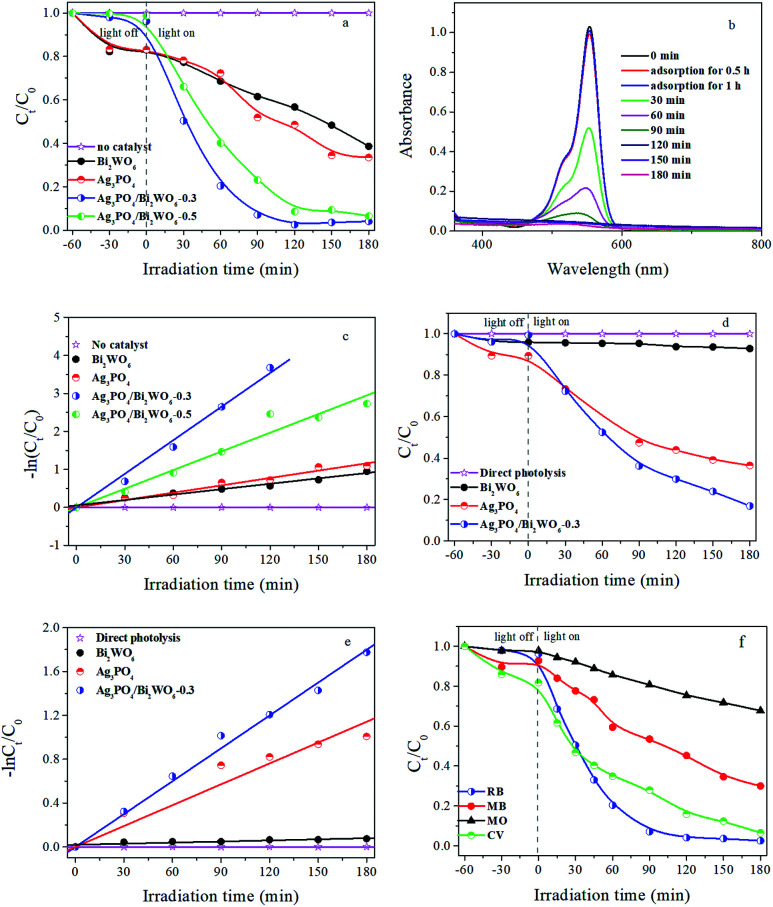
Photocatalytic activities of different photocatalysts toward (a) RB, (d) Phen, and (f) different organic dyes under visible-light irradiation. (b) UV-vis absorption spectra of the RB solution separated from Ag_3_PO_4_/Bi_2_WO_6_-0.3 suspension under visible-light illumination. Kinetic fitting curves for photocatalytic degradation of (c) RB and (e) Phen over different photocatalysts under visible-light irradiation.

Based on the above results, the photocatalytic activities of prepared photocatalysts were evaluated by degradation of RB under visible-light irradiation. As shown in Fig. 7a, adsorption tests display all suspensions of photocatalyst and RB reach adsorption–desorption equilibrium after 1 h stirring in dark. Meanwhile, the visible-light photocatalytic activities of samples were investigated. The results show that: (1) without the catalyst, no RB is degraded under visible-light irradiation for 180 min, implying RB is relative stability under longtime irradiation; (2) in the presence of catalysts and light, the RB-degradation efficiency is greatly improved. Compared with pure Bi_2_WO_6_ and Ag_3_PO_4_, Ag_3_PO_4_/Bi_2_WO_6_ composites show higher photocatalytic activity. Furthermore, with increasing molar ratio of Ag_3_PO_4_/Bi_2_WO_6_ from 0 to 0.3, Ag_3_PO_4_/Bi_2_WO_6_ composites display enhanced photocatalytic activity and then decrease with increasing to 0.5. For example, RB-degradation efficiency of Bi_2_WO_6_, Ag_3_PO_4_, Ag_3_PO_4_/Bi_2_WO_6_-0.3, and Ag_3_PO_4_/Bi_2_WO_6_-0.5 can reach 43.2%, 51.4%, 97.5%, and 91.4% after 120 min visible-light illumination.

Fig. 7b shows the absorption spectra of RB (*λ*_max_ = 553 nm) under visible-light irradiating the photoactive Ag_3_PO_4_/Bi_2_WO_6_-0.3. The absorption peak intensity of RB located at 553 nm reduces rapidly with prolonged irradiation time, and it is hardly observed after 120 min visible-light illumination. The results are consistent with Fig. 7a. In addition, we find the obvious blue shift of the absorption peak at 553 nm, which corresponds to the de-ethylation process. Therefore, the cleavage of the whole conjugated chromophore structure of RB leads to rapidly reducing the absorption peak intensity of RB, which indicates intermediate products were formed and then degraded to small molecules or CO_2_ during the RB degradation process.^15,46^

The pseudo-first-order model based on the Langmuir–Hinshelwood (LH) kinetic model is mainly used to estimate the kinetics of the photocatalytic degradation of RB, as shown in the following equation:

4 −ln(*C*_*t*_/*C*_0_) = *k*_app_*t,*

where *k* is the apparent rate constant of degradation; *C*_*t*_ and *C*_0_ are the initial and instantaneous concentration of RB at the time *t*_0_ and *t*, respectively. Plots of −ln(*C*_*t*_/*C*_0_) *vs.* time for photocatalysts are shown in Fig. 7c. All these photocatalysts display good linear relation meeting a pseudo-first-order reaction, and the kinetic constants calculated are 0 min^−1^ (no photocatalyst), 0.00515 min^−1^ (Bi_2_WO_6_), 0.00646 min^−1^ (Ag_3_PO_4_), 0.0295 min^−1^ (Ag_3_PO_4_/Bi_2_WO_6_-0.3), and 0.0164 min^−1^ (Ag_3_PO_4_/Bi_2_WO_6_-0.5), respectively. During all photocatalysts, Ag_3_PO_4_/Bi_2_WO_6_-0.3 shows the highest photocatalytic activity and decomposition rate, which are 5.7 and 4.6 times than that of Bi_2_WO_6_ and Ag_3_PO_4_, respectively. In short, Ag_3_PO_4_/Bi_2_WO_6_-0.3 exhibits the dramatical enhancement on photocatalytic activity of identical conditions than pure Bi_2_WO_6_ and Ag_3_PO_4_.

In order to eliminate the influence of dye sensitization, Phen as the colorless compound is selected as a model molecule to further study the photocatalytic performance of Ag_3_PO_4_/Bi_2_WO_6_ under visible-light irradiation. Because that Phen (*λ*_max_ = 270 nm) has no absorption and no photosensitization in visible-light region. Fig. 7d and e show the relative concentration (*C*_*t*_/*C*_0_) and −ln(*C*_*t*_/*C*_0_) *vs.* time curves of Phen in the presence of Bi_2_WO_6_, Ag_3_PO_4_, and Ag_3_PO_4_/Bi_2_WO_6_-0.3. As displayed in Fig. 7d, the direct photolysis of Phen is neglected during the whole visible-light irradiation, indicating Phen is also a stable pollutant. However, the degradation efficiency of Phen is about 7.1%, 63.5%, and 83.1% in the presence of Bi_2_WO_6_, Ag_3_PO_4_, and Ag_3_PO_4_/Bi_2_WO_6_-0.3, respectively. Their corresponding rate constants are obtained from Fig. 7e to be 0.00033 min^−1^, 0.00634 min^−1^, and 0.00999 min^−1^, respectively. The results are consistent with those obtained for RB degradation. Meanwhile, the above results confirm that photocatalytic performance of Ag_3_PO_4_/Bi_2_WO_6_-0.3 is due to the excitation of the photocatalyst rather than the sensitization mechanism.

In order to test the extensive adaptability of the Ag_3_PO_4_/Bi_2_WO_6_ photocatalyst, anionic dye (MO) and cationic dyes (RB, MB, and CV) are also employed as target pollutants. As depicted in Fig. 7f, MO, RB, MB, and CV are effectively eliminated after visible-light irradiation, which implies that the Ag_3_PO_4_/Bi_2_WO_6_ photocatalyst is highly efficient in the visible-light degradation of pollutants, especially cationic dyes. The excellent photocatalytic activity of Ag_3_PO_4_/Bi_2_WO_6_ towards to organic pollutants is due to the good formation of heterostructures, which not only broaden the spectral response range to visible light but also effectively promote the charge separation. In addition, the excessive Ag_3_PO_4_ may act as a recombination center, and cover the active sites on the Bi_2_WO_6_ surface, leading to reducing the separation efficiency of the photogenerated charge carriers. It can be confirmed from the following PL results (Fig. S4, ESI†). That is why Ag_3_PO_4_/Bi_2_WO_6_-0.3 shows higher photocatalytic activity than Ag_3_PO_4_/Bi_2_WO_6_-0.5.

**Fig. 8:**
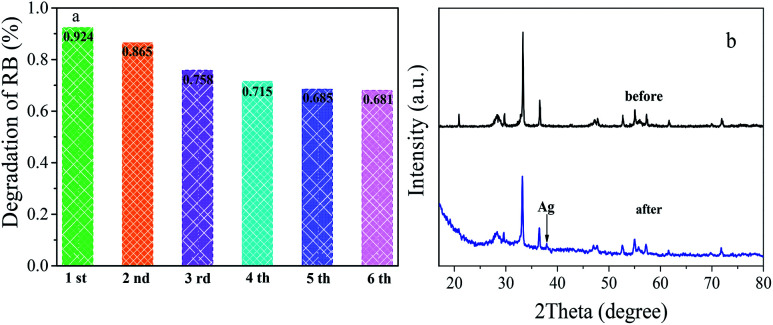
Cyclic photocatalytic degradation of RB over Ag_3_PO_4_/Bi_2_WO_6_-0.3 (a); XRD patterns of Ag_3_PO_4_/Bi_2_WO_6_-0.3 before and after the cycle degradation experiments (b).

The photostability of a photocatalyst is essential for practical application. To research the reusability of photocatalysts, recycled photocatalytic degradation RB test was performed over Ag_3_PO_4_/Bi_2_WO_6_-0.3 composite. As shown in Fig. 8a, the photodegradation percentage of RB after visible light irradiation for 90 min reduced from the original 92.9–86.5%, 75.8%, 71.5%, 68.5% and 68.1% after six cycles. Although the degradation efficiency of RB in the reactive system went on 24.8%, the photodegradation percentage of RB was almost the same after the five and six cycles, indicating that the catalyst had a certain stability. The slight decline of photocatalytic efficiency is attributed to the inevitable loss of photocatalysts during the recycle runs. In addition, the color of RB-photocatalysts suspension had been changed from pink to black, indicating metallic Ag was formed on the surface of the catalyst during the photocatalytic process. This phenomenon is confirmed by the comparative XRD patterns of Ag_3_PO_4_/Bi_2_WO_6_ before and after the photocatalytic experiments in Fig. 8b. There is one weak peak located at 38.1° for Ag_3_PO_4_/Bi_2_WO_6_-0.3 after cycles, which can be classified as the characteristic peak of metallic silver.^47^ This part Ag formed at the early photocatalytic reaction. Meanwhile, UV-vis/DRS, the high-resolution XPS spectra of Ag 3d, and SEM image of Ag_3_PO_4_/Bi_2_WO_6_-0.3 after reaction are also shown in Fig. S3 (ESI†). As displayed in Fig. S3(a),† Ag_3_PO_4_/Bi_2_WO_6_-0.3 displays enhanced photo-absorption in the visible-light region after the cycle degradation experiments. In particular, a broad prominent absorption in the visible-light region of 450–800 nm is observed, owing to the surface plasmon resonance (SPR) effect of Ag nanoparticles.^47^ Fig. S3(b)† shows the Ag XPS spectra of Ag_3_PO_4_/Bi_2_WO_6_-0.3 after cycles. The peaks located at 368.8 and 374.9 eV are assigned to Ag^0^, and the typical peaks located at 367.8 and 373.8 eV are ascribed to Ag^+^. According to the XPS results, the content of Ag^0^ is about 30% after cycles. As shown in Fig. S3(c),† although the aggregation phenomenon becomes more obvious after the photocatalytic reaction, Ag_3_PO_4_/Bi_2_WO_6_-0.3 still maintain their morphologies. These results further prove that the photocorrosion resistance and stability of Ag_3_PO_4_ were improved by introducing Bi_2_WO_6_ to construct Ag_3_PO_4_-Ag-Bi_2_WO_6_ Z-scheme heterostructures.

### 3.4 Photocatalytic mechanism discussion

#### 3.4.1 Free radical and hole scavenging experiments

According to the photocatalytic literature,^48,49^ the movement of the charge carriers such as e^−^ and h^+^ is the crucial parameter to generate reactive species such as ˙O^2−^, ˙OH, and h^+^. Thus, active species generated during the photodegradation process of RB over the Ag_3_PO_4_/Bi_2_WO_6_-0.3 are identified by free radical and hole trapping experiments in the presence of various scavengers such as *tert*-butyl alcohol (*t*-BuOH, ˙OH scavenger, 1 mM), ethylenediaminetetraacetic acid disodium salt (EDTA-2Na, h^+^ scavenger, 1 mM), and benzoquinone(BQ, ˙O^2−^ scavenger, 1 mM).

**Fig. 9:**
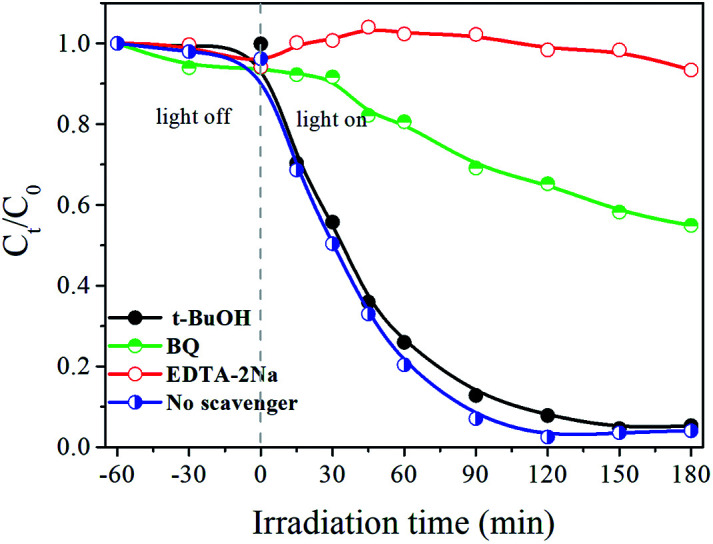
Effects of different scavengers on the photodegradation of RB over the Ag_3_PO_4_/Bi_2_WO_6_-0.3 composite.

Fig. 9 depicts the effects of various scavengers on the degradation of RB. The addition of *t*-BuOH can hardly inhibit RB degradation, which demonstrates that hydroxyl radicals ˙OH have little influence in the photocatalytic process. However, when the EDTA-2Na and BQ were added into the reaction system, the photocatalytic activities of Ag_3_PO_4_/Bi_2_WO_6_-0.3 are obviously decelerated. Furthermore, the photodegradation percentage of RB is reduced from the original 100–6.6% and 45.1%, respectively. The above results suggested that h^+^ and ˙O^2−^ are main active species during the degradation process.

#### 3.4.2 Photoelectrochemical experiments

**Fig. 10:**
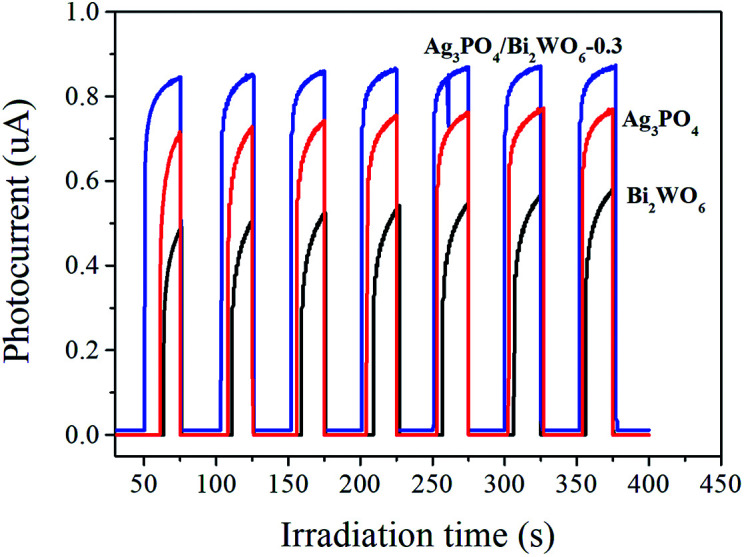
Photocurrent responses of Ag_3_PO_4_, Bi_2_WO_6_, and Ag_3_PO_4_/Bi_2_WO_6_-0.3 under visible light irradiation.

Photoelectrochemical experiments as a useful technique to monitor the generation of photoelectrons and holes as well as their transfer and efficient separation in the Ag_3_PO_4_/Bi_2_WO_6_ system were performed. As shown in Fig. 10, the transient photocurrent responses of Bi_2_WO_6_, Ag_3_PO_4_, and Ag_3_PO_4_/Bi_2_WO_6_-0.3 are recorded for several on–off cycles under visible-light irradiation at a bias of 1 V. It is found that the photocurrent responses of the tested sample working electrodes decrease to zero as soon as the lamp is turned off, and a rapid increase appears when the light is on. Meanwhile, photocurrent responses regain a reproducible value when the lamp is turned on again during on–off intermittent irradiation cycles. In addition, the Ag_3_PO_4_/Bi_2_WO_6_-0.3 exhibits the enhanced the photocurrent compared with Bi_2_WO_6_ and Ag_3_PO_4_. The sequence of the photocurrent is in accordance with the order of the photocatalytic activity of photocatalysts. This suggests a smaller recombination and a more efficient separation of photogenerated e^−^–h^+^ pairs occurs across the interface between Bi_2_WO_6_ and Ag_3_PO_4_ in the Ag_3_PO_4_/Bi_2_WO_6_ composite.

**Fig. 11:**
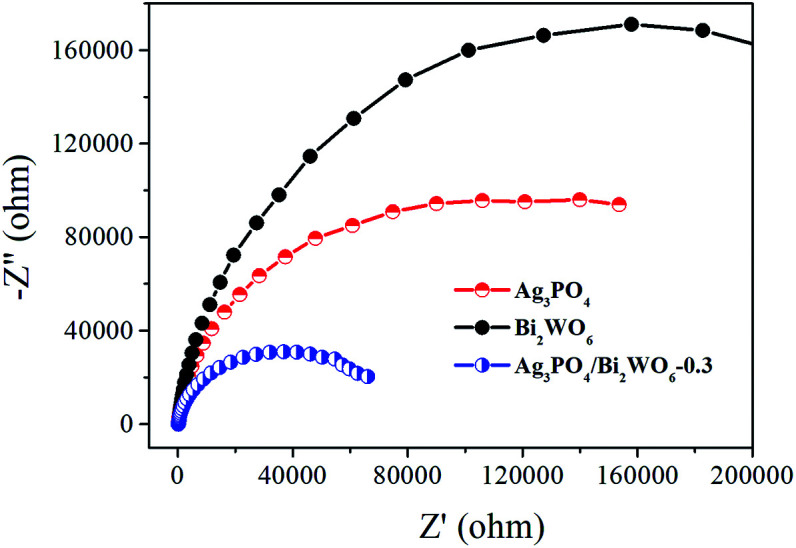
EIS Nyquist plots of Ag_3_PO_4_, Bi_2_WO_6_, and Ag_3_PO_4_/Bi_2_WO_6_-0.3.

Electrochemical impedance spectroscopy (EIS) is also employed to investigate the charge transfer resistance and the separation of photogenerated e^−^–h^+^ pairs at solid/electrolyte interfaces in the photocatalyst.^50^Fig. 11 displays the EIS Nyquist plots of Bi_2_WO_6_, Ag_3_PO_4_, and Ag_3_PO_4_/Bi_2_WO_6_-0.3 under visible-light irradiation. It is clearly seen that the smallest arc radius of the EIS Nyquist plot of Ag_3_PO_4_/Bi_2_WO_6_-0.3 implies that it has the fastest interfacial electron transfer and more separation of photogenerated e^−^–h^+^ pairs when compared to those of Bi_2_WO_6_ and Ag_3_PO_4_. It is the reason for the Ag_3_PO_4_/Bi_2_WO_6_-0.3 composites exhibit the highest photocatalytic activity.

**Scheme 1:**
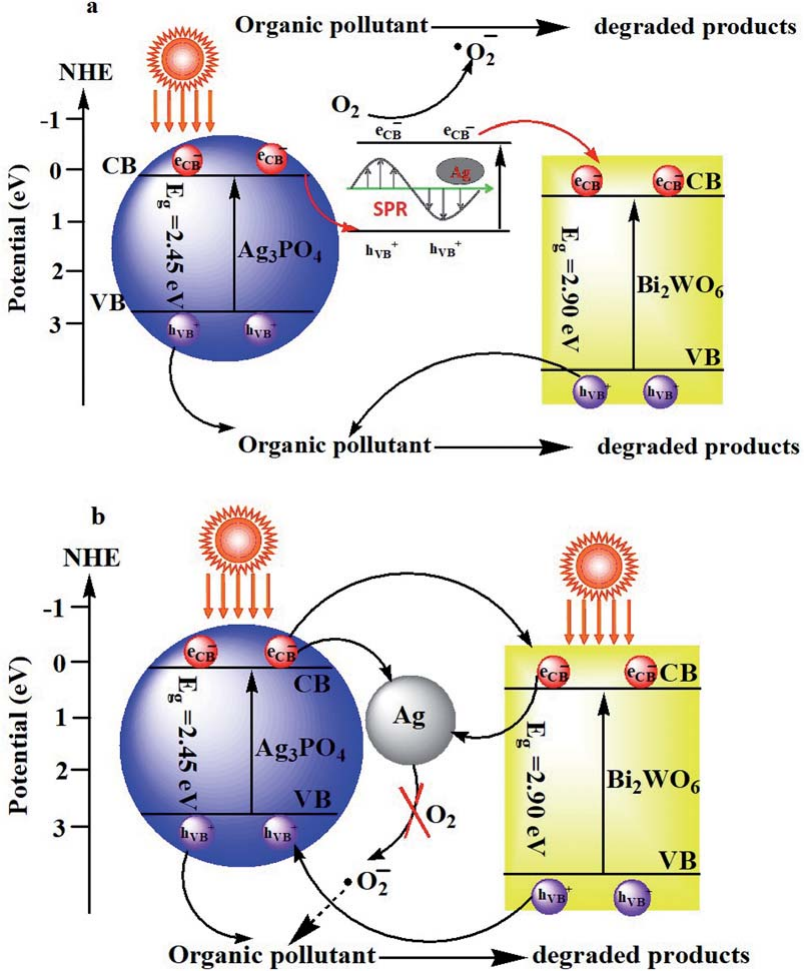
schematic diagram of the separation and transfer of photogenerated charges in the Ag_3_PO_4_/Bi_2_WO_6_ heterostructures based on the plasmonic Z-scheme theory (a) and heterojunction energy-band theory (b) under visible light irradiation.

Photoluminescence (PL) spectrum is also an effective tool to reveal the migration, transfer and recombination processes of the photogenerated e^−^–h^+^ pairs in semiconductors. Usually, a lower PL intensity indicates lower recombination rate of the charge carriers and higher photocatalytic activities of the photocatalysts. Fig. S4 (ESI†) shows the PL spectra of as-prepared Ag_3_PO_4_, Bi_2_WO_6_, and Ag_3_PO_4_/Bi_2_WO_6_ composites with an excitation wavelength of 360 nm. As for the Bi_2_WO_6_-based materials, their PL intensities follow the order Bi_2_WO_6_ > Ag_3_PO_4_/Bi_2_WO_6_-0.5 > Ag_3_PO_4_/Bi_2_WO_6_-0.3 > Ag_3_PO_4_. Compared with pure Bi_2_WO_6_, the PL intensities of Ag_3_PO_4_/Bi_2_WO_6_ composites present an obvious decrease, implying a lower recombination feasibility of free charges in the Ag_3_PO_4_/Bi_2_WO_6_ heterostructures. The Ag_3_PO_4_/Bi_2_WO_6_ nanocomposite displays a higher PL intensity than Ag_3_PO_4_, implying that the migration pathways of the photoexcited e^−^–h^+^ in the Ag_3_PO_4_/Bi_2_WO_6_ nanocomposite is plasmonic Z-scheme theory (Scheme 1a) not as heterojunction energy-band theory (Scheme 1b).^51,52^

From the Scheme 1a, under visible-light irradiation, both Bi_2_WO_6_ and Ag_3_PO_4_ can be excited to produce e^−^ and h^+^ simultaneously owing to their visible-light response. On the one hand, the part of Ag_3_PO_4_ can be photoreduced to Ag^0^ during the initial photocatalytic process. Ag nanoparticles (NPs) can absorb visible light and induce e^−^–h^+^ pairs on account of the dipolar character and the SPR effect of the Ag NPs. The photogenerated electrons in the CB of Ag_3_PO_4_ shift to the photogenerated holes produced by plasmonic absorption in the Ag NPs, which results in higher PL intensity. On other the hand, the plasmon hot electrons generated by the localized SPR oscillations of Ag NPs can capture the dissolved O_2_ in water to form ˙O^2−^ (ref. 53). Meanwhile, the plasmon hot electrons directly transfer from the Ag NPs to the CB of Bi_2_WO_6_. While the photogenerated holes are still in the VB of Ag_3_PO_4_ and Bi_2_WO_6_. Therefore, the photo-generated charge carriers are efficiently separated in space, which retards the photocorrosion of Ag_3_PO_4_. With the assistance of h^+^ and ˙O^2−^ the two main active species, the organic pollutant is effectively degraded in aqueous solution. But from the heterojunction energy-band theory described in Scheme 1b, the VB potential (2.68 eV *vs.* NHE) of Ag_3_PO_4_ is more negative than that of Bi_2_WO_6_ (3.08 eV *vs.* NHE), which leads to the migration of the h^+^ from Bi_2_WO_6_ to Ag_3_PO_4_. Since the CB potential (0.24 eV *vs.* NHE) of Ag_3_PO_4_ is more negative than that of Bi_2_WO_6_ (0.44 eV *vs.* NHE), photo-induced electrons from Ag_3_PO_4_ migrate to the CB of Bi_2_WO_6_ and then transfer to Ag^0^. Therefore, the photo-generated electrons cannot reduce O_2_ to produce ˙O^2−^, due to the CB potential of Bi_2_WO_6_ being more positive than the redox potential of ˙O^2−^ formation (O_2_/˙O^2−^ = −0.33 eV, NHE).^51^ Thus this phenomenon cannot explain the stronger effect of ˙O^2−^ on the degradation of RB. In conclusion, the plasmonic Z-scheme theory for the photocatalysis of Ag_3_PO_4_/Bi_2_WO_6_ is much more reasonable.

## 4. Conclusions

Novel Ag_3_PO_4_/Bi_2_WO_6_ heterostructured materials were successfully synthesized by assembling Ag_3_PO_4_ irregular nanospheres on the surface of Bi_2_WO_6_ nanoflakes. Ag_3_PO_4_/Bi_2_WO_6_-0.3 showed obviously superior visible-light catalytic activity toward degradation of organic pollutants. Free radical and hole scavenging experiments suggested h^+^ and ˙O^2−^ are two main active species through the degradation process. In brief, the enhanced photocatalytic activity was due to the good formation of heterostructures, which could not only broaden the spectral response range to visible light but also effectively promoted the charge separation. These results were solidly confirmed by photocurrent responses, electrochemical impedance spectroscopy, and photoluminescence spectrum. In addition, Ag_3_PO_4_/Bi_2_WO_6_-0.3 exhibited relative higher photostability toward RB degradation, which was explained by the reasonable photoreactive mechanism. This work provides the potential application of Bi_2_WO_6_-based heterostructures as efficient visible light responsive catalysts for environmental remediation.

## Conflicts of interest

There are no conflicts to declare.

## Supplementary Material

RA-008-C8RA01477A-s001
