# Supplementary material for: Enhanced visible-light photocatalytic activity and photostability of Ag3PO4/Bi2WO6 heterostructures toward organic pollutant degradation and plasmonic Z-scheme mechanism
Source: RSC Adv. 2018 Apr 27;8(28):15853–62. doi: 10.1039/c8ra01477a (PMC9080155; doi:10.1039/c8ra01477a)
Supplement: RA-008-C8RA01477A-s001 [file RA-008-C8RA01477A-s001.pdf]

Electronic Supplementary Material (ESI) for RSC Advances.
This journal is © The Royal Society of Chemistry 2018

Electronic Supplementary Information

# Enhanced visible light photocatalytic activity and photostability of $Ag_3PO_4/Bi_2WO_6$ heterostructures toward organic pollutants degradation and plasmonic Z-scheme mechanism

Fengyan Ma[a], Qilin Yang[a], Zhengjun Wang[a], Yahong Liu[a], Jianjiao Xin[a], Jingjing Zhang[a], Yuting Hao[a], and Li Li[a,b*]

[a]*College of Chemistry and Chemical Engineering, Qiqihar University, Qiqihar 161006, Heilongjiang, PR China*

[b]*College of Materials Science and Engineering, Qiqihar University, Qiqihar 161006, Heilongjiang, PR China*

---

* Corresponding authors.

*E-mail*: qqhrlili@126.com; Tel: +86-0452-2738206

Table 1. Comparison of photocatalytic activities of $Ag_3PO_4$-based photocatalysts.

| Catalyst | Light source/W | Dye | $C_{Dye}$/ppm | $V_{Dye}$/mL | Efficiency/Time | Ref |
|---|---|---|---|---|---|---|
| $Ag_3PO_4/Bi_2WO_6$ | Xe lamp/300 | RB | 50 | 220 | 92.7%/90 min | This work |
| $Ag_3PO_4$/ZnO-IO | Xe lamp/300 | RB | 5 | 60 | 32.0%/240 min | 25 |
| $Ag_3PO_4$/GO | tungsten halogen lamp/500W | RB | 40 | 200 | 100%/300 min | 26 |
| $Ag_3PO_4/InVO_4/BiVO_4$ | Xe lamp/300 | RB | 5 | 100 | 100%/120 min | 27 |
| $Ag/Ag_3PO_4/Bi_2MoO_6$ | Xe lamp/300 | RB | 5 | 100 | 100%/80 min | 28 |
| $Ag_3PO_4/Bi_2WO_6$ | Xe lamp | RB | 5 | 200 | 100%/80 min | 32 |

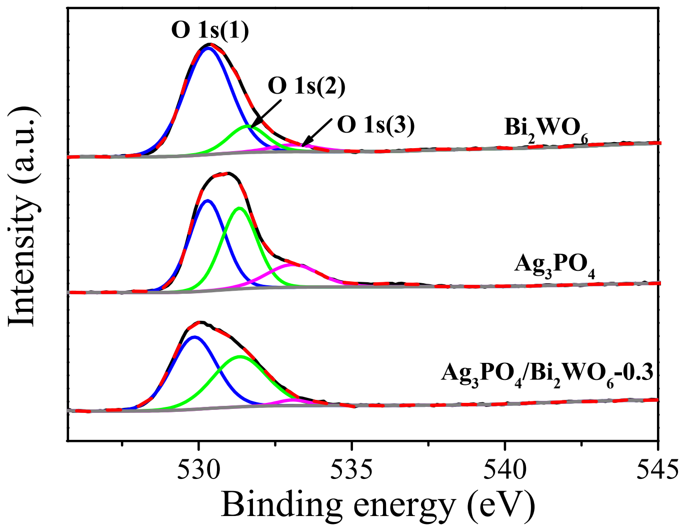


Fig. S1 The high-resolution XPS spectra of O 1s for the $Bi_2WO_6$, $Ag_3PO_4$, and $Ag_3PO_4/Bi_2WO_6$-0.3.

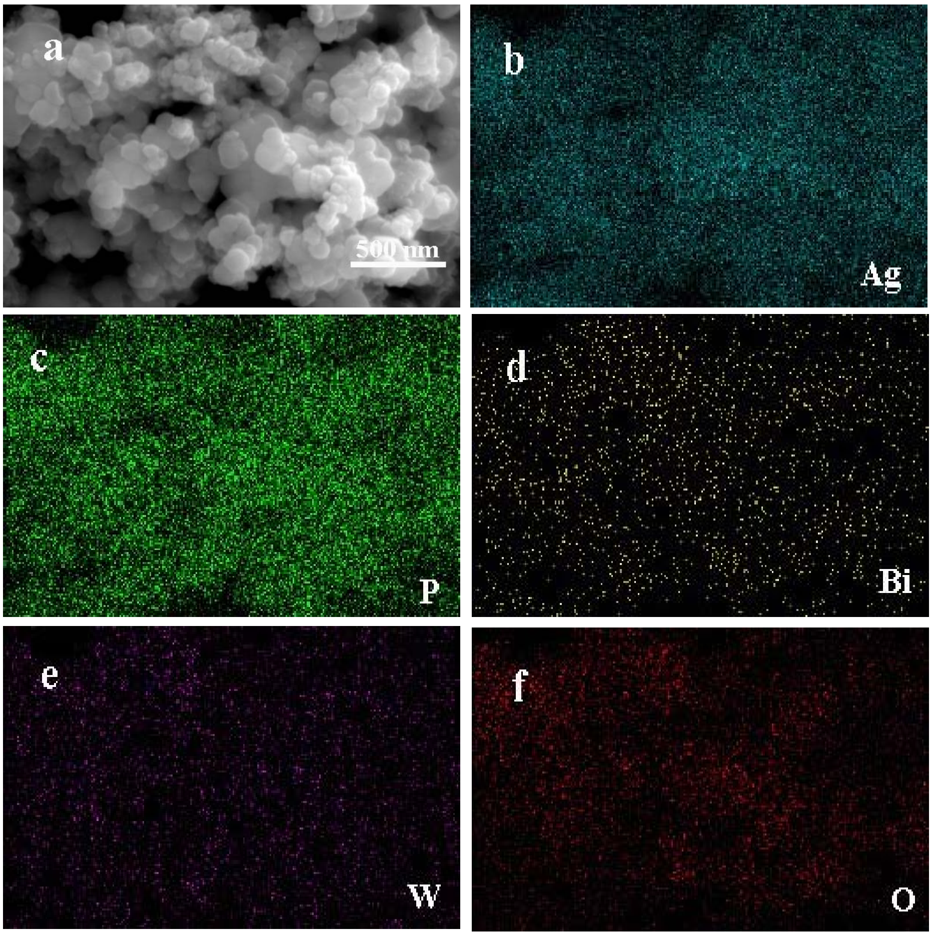


Fig. S2 SEM image (a) and EDS element mapping images of Ag (b), P (c), Bi (d), W (e), and O (f) of the as-obtained $Ag_3PO_4/Bi_2WO_6$ heterostructures.

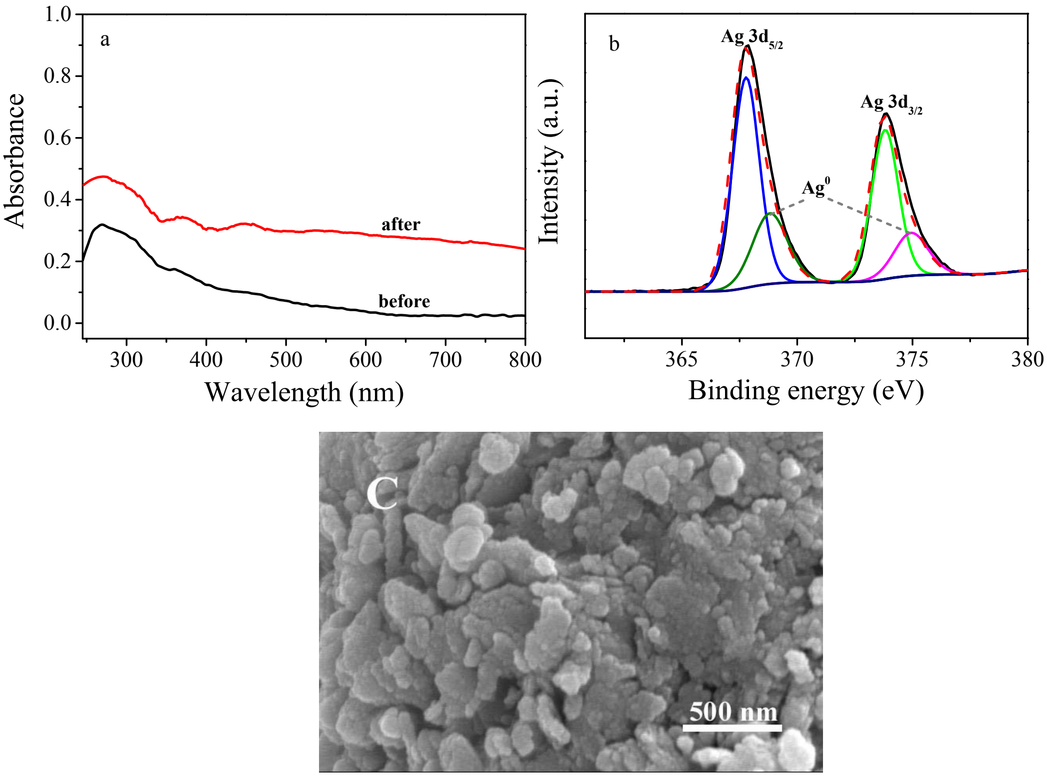


Fig. S3 UV-vis/DRS (a), the high-resolution XPS spectra of Ag 3d (b), and SEM image of $Ag_3PO_4/Bi_2WO_6$-0.3 (c) after the cycle degradation experiments.

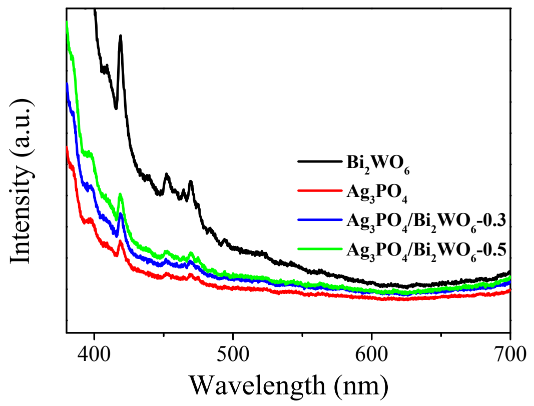


Fig. S4 Photoluminescence (PL) spectra of as-prepared $Ag_3PO_4$, $Bi_2WO_6$, and $Ag_3PO_4/Bi_2WO_6$ material.
